# *catena*-Poly[[tetra­kis(hexa­methyl­phospho­ramide-κ*O*)bis­(nitrato-κ^2^ *O*,*O*′)lanthanum(III)] [silver(I)-di-μ_2_-sulfido-tungstate(VI)-di-μ_2_-sulfido]]

**DOI:** 10.1107/S1600536807066597

**Published:** 2008-01-23

**Authors:** Guodong Tang, Jinfang Zhang, Chi Zhang, Lude Lu

**Affiliations:** a School of Chemical Engineering, Nanjing University of Science and Technology, 200 Xiaolingwei, Nanjing 210094, Jiangsu, People’s Republic of China; b Huaian Teachers College, Huaian 223001, Jiangsu People’s Republic of China

## Abstract

Hexamethyl­phospho­ramide (hmp), tetra­thio­tungstate(VI), silver sulfide and lanthanum(III) nitrate are self-assembled to form discrete cations one-dimensional poylmeric anionic chains [AgWS_4_]*_n_^n^* ^−^ in the title compound, {[La(NO_3_)_2_(C_6_H_18_N_3_OP)_4_][AgWS_4_]}_*n*_. The central La atom in the cation is coordinated by eight O atoms from two nitrate and four hmp ligands. Together with the two nitrate ligands, the cation is monovalent, which leads to the anionic chain having a monovalent repeat unit. The polymeric anionic chain with W⋯Ag⋯W and Ag⋯W⋯Ag angles of 165.94 (3) and 155.894 (14)° presents a distorted linear configuration. Five N atoms, 18 C atoms and their attached H atoms are disordered equally over two positions.

## Related literature

The one-dimensional W/S/Ag anionic polymers {(*γ*-Me­PyH)[WS_4_Ag]}_*n*_ (Lang *et al.*, 1993 ▶) and {[NH_3_C(CH_2_OH)_3_][WS_4_Ag](2DMF)}_*n*_ (Huang *et al.*, 1997 ▶) have ideal and nearly linear configurations,  respectively. Two analogs of the title compound, {[Eu(hmp)_4_(NO_3_)_2_][WS_4_Ag]}_*n*_ (Zhang, Qian *et al.*, 2007 ▶) and {[Y(hmp)_4_(NO_3_)_2_][WS_4_Ag]}_*n*_ (Zhang, Cao *et al.*, 2007 ▶), have similar wave-like chains. {[Nd(dmf)_8_][W_4_S_16_Ag_5_]}_*n*_ (Huang *et al.*, 1996 ▶) has solvent-coordinated rare-earth cations leading to an anionic chain with a trivalent repeat unit. For a review of polymeric Mo(W)/S/Ag(Cu) clusters, see: Niu *et al.* (2004 ▶). For a review of the third-order non-linear optical properties of Mo(W)/S/Ag(Cu) clusters, see: Zhang, Song *et al.* (2007 ▶).
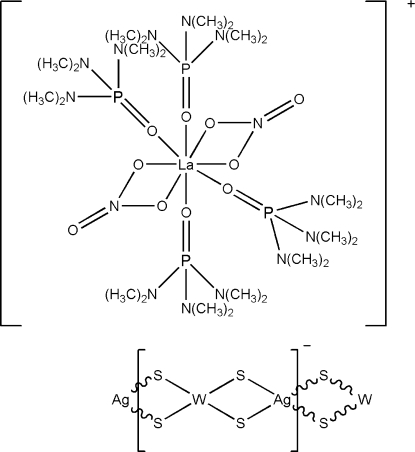


## Experimental

### 

#### Crystal data

[La(NO_3_)_2_(C_6_H_18_N_3_OP)_4_][AgWS_4_]
*M* *_r_* = 1399.71
Monoclinic, 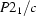

*a* = 15.883 (2) Å
*b* = 30.070 (4) Å
*c* = 11.5283 (15) Å
β = 90.502 (3)°
*V* = 5505.8 (12) Å^3^
*Z* = 4
Mo *K*α radiation
μ = 3.52 mm^−1^
*T* = 295 (2) K
0.50 × 0.42 × 0.38 mm

#### Data collection

Rigaku Mercury diffractometer
Absorption correction: multi-scan (*SADABS*; Sheldrick, 1996 ▶) *T*_min_ = 0.18, *T*_max_ = 0.26
50056 measured reflections
10031 independent reflections
8984 reflections with *I* > 2σ(*I*)
*R*_int_ = 0.049

#### Refinement

*R*[*F* ^2^ > 2σ(*F* ^2^)] = 0.059
*wR*(*F* ^2^) = 0.137
*S* = 1.15
10031 reflections
462 parameters
32 restraints
H-atom parameters constrained
Δρ_max_ = 1.07 e Å^−3^
Δρ_min_ = −1.90 e Å^−3^

### 

Data collection: *CrystalClear* (Rigaku, 2000 ▶); cell refinement: *CrystalClear*; data reduction: *CrystalStructure* (Rigaku/MSC, 2002 ▶); program(s) used to solve structure: *SHELXS97* (Sheldrick, 1997 ▶); program(s) used to refine structure: *SHELXL97* (Sheldrick, 1997 ▶); molecular graphics: *SHELXL97*; software used to prepare material for publication: *SHELXL97*.

## Supplementary Material

Crystal structure: contains datablocks I, global. DOI: 10.1107/S1600536807066597/hg2364sup1.cif

Structure factors: contains datablocks I. DOI: 10.1107/S1600536807066597/hg2364Isup2.hkl

Additional supplementary materials:  crystallographic information; 3D view; checkCIF report

## Figures and Tables

**Fig. 1.**
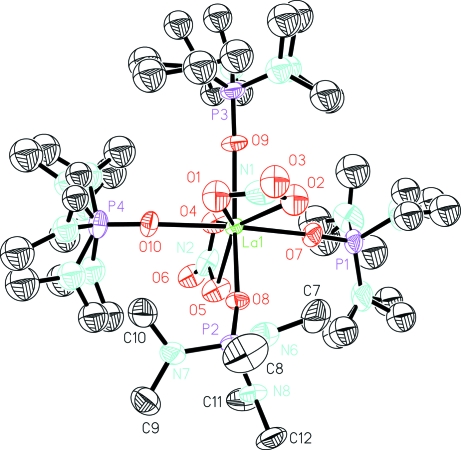
The molecular structure of the cation in the title compound, with atom labels and 30% probability displacement ellipsoids. All H atoms have been omitted.

**Fig. 2.**
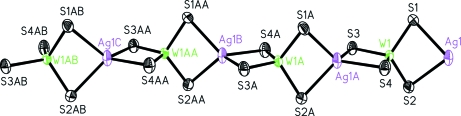
The molecular structure of a portion of the anionic chain in the title compound, with atom labels and 30% probability displacement ellipsoids. (symmetry codes: A x, -y + 1/2, z + 1/2; AA and B x, y, z + 1; AB and C x, -y + 1/2, z + 1.5).

## supplementary crystallographic information

### Comment

One-dimensional Mo(W)/S/Ag anionic polymers have attracted much attention for their configurational isomerism (Niu *et al.*, 2004) and unique properties as functional materials, such as third-order nonlinear optical (NLO) materials (Zhang, Song *et al.*, 2007, and references therein). Different solvent-coordinated rare-earth cations proved effective to obtain various configurations of anionic chains (Niu *et al.*, 2004). The title compound {[La(hmp)_4_(NO_3_)_2_][WS_4_Ag]}_n_ (hmp = hexamethylphosphoramide) with a wave-like anionic chain was prepared by following such route using La(III)-hmp complex as counterion.

The cation in the title compound, where La^3-^ is coordinated by eight O atoms from two nitrate and four hmp ligands, has the same structure as those in the isostructural {[Eu(hmp)_4_(NO_3_)_2_][WS_4_Ag]}_n_ (Zhang, Qian *et al.*, 2007) and {[Y(hmp)_4_(NO_3_)_2_][WS_4_Ag]}_n_ (Zhang, Cao *et al.*, 2007). Parts of dimethylamine groups from hmp ligands have large librations, which gives rise to some disordered C and N atoms. In possession of two nitrate ligands, the cation in the title compound is univalent (Fig. 1), which leads to an anionic chain with a univalent repeat unit, unlike other solvent-coordinated rare-earth cations (Niu *et al.*, 2004, and references therein), which are trivalent and induce trivalent repeat units. For example, [Nd(dmf)_8_]^3+^ induces an anionic chain with a trivalent repeat unit [W_4_S_16_Ag_5_]^3-^ (Huang *et al.*, 1996).

As illustrated in Fig. 2, the anionic chain in the title compound has a distorted linear configuration with W—Ag—W and Ag—W—Ag angles of 165.94 (3) and 155.894 (14) °, unlike those in {(*γ*-MePyH)[WS_4_Ag]}_n_ (Lang *et al.* 1993) and {[NH_3_C(CH_2_OH)_3_][WS_4_Ag](2DMF)}_n_ (Huang *et al.*, 1997), showing an ideal linear chain and a nearly linear chain, respectively. This fact suggests that cations with bigger bulk lead to more distorted anionic chains.

Similar angles for W—Ag—W and Ag—W—Ag are found in another two distorted linear chains in {[Eu(hmp)_4_(NO_3_)_2_][WS_4_Ag]}_n_ (Zhang, Qian *et al.*, 2007) and {[Y(hmp)_4_(NO_3_)_2_][WS_4_Ag]}_n_ (Zhang, Cao *et al.*, 2007), implying that different rare earth cations with the same coordination environments will result in the same anionic structures.

### Experimental

1 mmol A g_2_S was added to a solution of [NH_4_]_2_WS_4_ (2 mmol in 30 mL h mp) with thorough stirring for 9 h. The solution underwent an additional stir for one minute after 1 mmol La(NO_3_)_3_.6H_2_O was added. After filtration the orange-red filtrate was carefully laid on the surface with 30 ml *i*-PrOH. Orange-red block crystals were obtained after ten days. Yield: 1.302 g in pure form, 46.5% (based on W). Analysis calculated for C_24_H_72_AgLaN_14_O_10_P_4_S_4_W: C 20.59, H 5.18, N 14.01%; found: C 20.57, H 5.15, N 14.03%. IR: ν, cm^-1^, 482.9 m, 446.8 s (W-µ_2_-S).

### Refinement

H atoms were positioned geometrically and refined with riding model, with *U*_iso_ = 1.5*U*_eq_ for methyl H atoms and 0.96 Å for C—H bonds. Parts of dimethylamine groups from hmp ligands have large librations, resulting in some disordered C and N atoms. The occupancy for the disordered C and N atoms were all refined to be 0.5:0.5.

### Crystal data

**Table:**

[La(NO_3_)_2_(C_6_H_18_N_3_OP)_4_][AgWS_4_]	*F*_000_ = 2784
*M**_r_* = 1399.71	*D*_x_ = 1.689 Mg m^−^^3^
Monoclinic, *P*2_1_/*c*	Mo *K*α radiation λ = 0.71070 Å
Hall symbol: -P 2ybc	Cell parameters from 19164 reflections
*a* = 15.883 (2) Å	θ = 3.1–25.3º
*b* = 30.070 (4) Å	µ = 3.52 mm^−^^1^
*c* = 11.5283 (15) Å	*T* = 295 (2) K
β = 90.502 (3)º	Block, orange–red
*V* = 5505.8 (12) Å^3^	0.50 × 0.42 × 0.38 mm
*Z* = 4	

### Data collection

**Table:**

Rigaku Mercury diffractometer	10031 independent reflections
Radiation source: fine-focus sealed tube	8984 reflections with *I* > 2σ(*I*)
Monochromator: graphite	*R*_int_ = 0.049
Detector resolution: 7.31 pixels mm^-1^	θ_max_ = 25.4º
*T* = 295(2) K	θ_min_ = 3.2º
ω scans	*h* = −18→19
Absorption correction: multi-scan(SADABS; Sheldrick, 1996)	*k* = −36→35
*T*_min_ = 0.18, *T*_max_ = 0.26	*l* = −13→13
50056 measured reflections	

### Refinement

**Table:**

Refinement on *F*^2^	Secondary atom site location: difference Fourier map
Least-squares matrix: full	Hydrogen site location: inferred from neighbouring sites
*R*[*F*^2^ > 2σ(*F*^2^)] = 0.059	H-atom parameters constrained
*wR*(*F*^2^) = 0.137	*w* = 1/[σ^2^(*F*_o_^2^) + (0.0507*P*)^2^ + 23.8085*P*] where *P* = (*F*_o_^2^ + 2*F*_c_^2^)/3
*S* = 1.15	(Δ/σ)_max_ = 0.003
10031 reflections	Δρ_max_ = 1.07 e Å^−^^3^
462 parameters	Δρ_min_ = −1.90 e Å^−^^3^
32 restraints	Extinction correction: none
Primary atom site location: structure-invariant direct methods	

### Special details

**Table:**

Geometry. All e.s.d.'s (except the e.s.d. in the dihedral angle between two l.s. planes) are estimated using the full covariance matrix. The cell e.s.d.'s are taken into account individually in the estimation of e.s.d.'s in distances, angles and torsion angles; correlations between e.s.d.'s in cell parameters are only used when they are defined by crystal symmetry. An approximate (isotropic) treatment of cell e.s.d.'s is used for estimating e.s.d.'s involving l.s. planes.
Refinement. Refinement of *F*^2^ against ALL reflections. The weighted *R*-factor *wR* and goodness of fit *S* are based on *F*^2^, conventional *R*-factors *R* are based on *F*, with *F* set to zero for negative *F*^2^. The threshold expression of *F*^2^ > σ(*F*^2^) is used only for calculating *R*-factors(gt) *etc*. and is not relevant to the choice of reflections for refinement. *R*-factors based on *F*^2^ are statistically about twice as large as those based on *F*, and *R*- factors based on ALL data will be even larger.

### Fractional atomic coordinates and isotropic or equivalent isotropic displacement parameters (Å^2^)

**Table:**

	*x*	*y*	*z*	*U*_iso_*/*U*_eq_	Occ. (<1)
W1	0.21336 (2)	0.229586 (11)	0.47656 (3)	0.04668 (12)	
La1	0.73680 (3)	0.416357 (14)	0.34214 (4)	0.04265 (13)	
Ag1	0.21350 (6)	0.23828 (3)	0.21889 (6)	0.0770 (2)	
S1	0.09980 (16)	0.21700 (11)	0.3727 (2)	0.0748 (7)	
S2	0.32665 (16)	0.21611 (11)	0.3745 (2)	0.0768 (8)	
S3	0.21282 (17)	0.18414 (8)	0.6261 (2)	0.0658 (6)	
S4	0.21416 (19)	0.30013 (8)	0.5270 (2)	0.0727 (7)	
P1	0.6905 (2)	0.52880 (9)	0.1910 (2)	0.0736 (7)	
P2	0.51582 (13)	0.36353 (8)	0.3300 (2)	0.0551 (5)	
P3	0.96668 (14)	0.40731 (9)	0.2457 (3)	0.0683 (7)	
P4	0.7965 (2)	0.34795 (10)	0.6129 (2)	0.0765 (8)	
O1	0.7508 (6)	0.3388 (3)	0.2430 (8)	0.0964 (14)	
O2	0.7254 (6)	0.3932 (3)	0.1255 (7)	0.0964 (14)	
O3	0.7253 (6)	0.3263 (3)	0.0613 (7)	0.0964 (14)	
O4	0.7954 (5)	0.4739 (3)	0.4923 (7)	0.0887 (13)	
O5	0.6637 (5)	0.4594 (3)	0.5105 (7)	0.0887 (13)	
O6	0.7271 (5)	0.4970 (3)	0.6433 (7)	0.0887 (13)	
O7	0.7072 (4)	0.48424 (18)	0.2374 (6)	0.0679 (17)	
O8	0.5952 (3)	0.38996 (19)	0.3351 (5)	0.0573 (14)	
O9	0.8827 (3)	0.4191 (2)	0.2935 (6)	0.0667 (17)	
O10	0.7731 (4)	0.3691 (2)	0.5022 (6)	0.0700 (17)	
N1	0.7346 (6)	0.3524 (4)	0.1427 (9)	0.090 (3)	
N2	0.7295 (7)	0.4776 (3)	0.5514 (8)	0.081 (3)	
N3	0.7556 (9)	0.5664 (3)	0.2417 (13)	0.128 (4)	
N4	0.7075 (8)	0.5308 (4)	0.0529 (8)	0.111 (4)	
N5	0.5964 (9)	0.5450 (4)	0.2264 (11)	0.147 (6)	
N6	0.5012 (5)	0.3417 (3)	0.2041 (8)	0.083 (3)	
N7	0.5174 (5)	0.3229 (3)	0.4237 (8)	0.081 (2)	
N8	0.4369 (4)	0.3963 (3)	0.3588 (7)	0.069 (2)	
N9	0.9891 (7)	0.3543 (5)	0.2371 (16)	0.149 (6)	
N10	1.0346 (11)	0.4398 (7)	0.2820 (17)	0.086 (4)*	0.50
N10'	1.0389 (12)	0.4211 (6)	0.3363 (17)	0.086 (4)*	0.50
N11	0.9609 (14)	0.4042 (7)	0.103 (2)	0.093 (4)*	0.50
N11'	0.9732 (14)	0.4250 (7)	0.1080 (19)	0.093 (4)*	0.50
N12	0.8974 (14)	0.3616 (7)	0.642 (2)	0.102 (5)*	0.50
N12'	0.8564 (14)	0.3715 (8)	0.700 (2)	0.102 (5)*	0.50
N13	0.7778 (12)	0.2976 (6)	0.6083 (16)	0.086 (4)*	0.50
N13'	0.8541 (12)	0.3015 (6)	0.5863 (15)	0.086 (4)*	0.50
N14	0.7403 (14)	0.3730 (8)	0.7122 (19)	0.108 (5)*	0.50
N14'	0.7166 (14)	0.3280 (8)	0.684 (2)	0.108 (5)*	0.50
C1	0.8422 (17)	0.5726 (16)	0.204 (4)	0.200 (10)*	0.50
H1A	0.8673	0.5967	0.2471	0.300*	0.50
H1B	0.8429	0.5793	0.1231	0.300*	0.50
H1C	0.8736	0.5458	0.2188	0.300*	0.50
C1'	0.780 (3)	0.576 (2)	0.358 (3)	0.200 (10)*	0.50
H1'A	0.8200	0.5999	0.3582	0.300*	0.50
H1'B	0.8049	0.5501	0.3921	0.300*	0.50
H1'C	0.7312	0.5846	0.4011	0.300*	0.50
C2	0.745 (3)	0.586 (2)	0.354 (3)	0.200 (10)*	0.50
H2A	0.7892	0.6072	0.3683	0.300*	0.50
H2B	0.7478	0.5631	0.4125	0.300*	0.50
H2C	0.6915	0.6006	0.3578	0.300*	0.50
C2'	0.716 (3)	0.6095 (10)	0.220 (4)	0.200 (10)*	0.50
H2'A	0.7507	0.6328	0.2510	0.300*	0.50
H2'B	0.6618	0.6103	0.2573	0.300*	0.50
H2'C	0.7083	0.6137	0.1382	0.300*	0.50
C3	0.7228 (19)	0.4955 (9)	−0.025 (2)	0.116 (7)*	0.50
H3A	0.7313	0.5072	−0.1017	0.175*	0.50
H3B	0.6752	0.4758	−0.0264	0.175*	0.50
H3C	0.7721	0.4794	−0.0008	0.175*	0.50
C3'	0.6771 (19)	0.4955 (9)	−0.018 (3)	0.116 (7)*	0.50
H3'A	0.6917	0.5011	−0.0975	0.175*	0.50
H3'B	0.6170	0.4936	−0.0117	0.175*	0.50
H3'C	0.7020	0.4680	0.0068	0.175*	0.50
C4	0.730 (3)	0.5756 (9)	0.013 (4)	0.154 (10)*	0.50
H4A	0.7398	0.5749	−0.0692	0.231*	0.50
H4B	0.7802	0.5853	0.0523	0.231*	0.50
H4C	0.6849	0.5958	0.0292	0.231*	0.50
C4'	0.701 (3)	0.5686 (10)	−0.027 (3)	0.154 (10)*	0.50
H4'A	0.7143	0.5591	−0.1043	0.231*	0.50
H4'B	0.7390	0.5916	−0.0034	0.231*	0.50
H4'C	0.6441	0.5799	−0.0265	0.231*	0.50
C5	0.546 (2)	0.5814 (10)	0.178 (3)	0.143 (9)*	0.50
H5A	0.4919	0.5818	0.2143	0.214*	0.50
H5B	0.5390	0.5771	0.0961	0.214*	0.50
H5C	0.5742	0.6091	0.1921	0.214*	0.50
C5'	0.588 (2)	0.5938 (7)	0.224 (3)	0.143 (9)*	0.50
H5'A	0.5323	0.6021	0.2455	0.214*	0.50
H5'B	0.5999	0.6045	0.1474	0.214*	0.50
H5'C	0.6280	0.6066	0.2780	0.214*	0.50
C6	0.533 (2)	0.5177 (13)	0.282 (4)	0.122 (7)*	0.50
H6A	0.4829	0.5351	0.2936	0.184*	0.50
H6B	0.5539	0.5073	0.3551	0.184*	0.50
H6C	0.5197	0.4928	0.2330	0.184*	0.50
C6'	0.532 (2)	0.5122 (13)	0.250 (4)	0.122 (7)*	0.50
H6'A	0.4807	0.5268	0.2687	0.184*	0.50
H6'B	0.5501	0.4938	0.3135	0.184*	0.50
H6'C	0.5240	0.4940	0.1820	0.184*	0.50
C7	0.5137 (9)	0.3685 (6)	0.1023 (12)	0.134 (6)	
H7A	0.5030	0.3509	0.0343	0.201*	
H7B	0.5707	0.3791	0.1012	0.201*	
H7C	0.4758	0.3934	0.1035	0.201*	
C8	0.4660 (10)	0.2969 (5)	0.1868 (15)	0.145 (7)	
H8A	0.4630	0.2904	0.1054	0.218*	
H8B	0.4105	0.2956	0.2191	0.218*	
H8C	0.5015	0.2754	0.2248	0.218*	
C9	0.4528 (8)	0.3133 (5)	0.5070 (11)	0.106 (4)	
H9A	0.4687	0.2878	0.5520	0.159*	
H9B	0.4007	0.3075	0.4671	0.159*	
H9C	0.4459	0.3384	0.5576	0.159*	
C10	0.5850 (8)	0.2909 (4)	0.4234 (13)	0.112 (4)	
H10A	0.5762	0.2695	0.4839	0.167*	
H10B	0.6376	0.3059	0.4364	0.167*	
H10C	0.5861	0.2760	0.3498	0.167*	
C11	0.4436 (7)	0.4301 (4)	0.4476 (12)	0.106 (4)	
H11A	0.3914	0.4461	0.4523	0.159*	
H11B	0.4881	0.4504	0.4284	0.159*	
H11C	0.4558	0.4164	0.5210	0.159*	
C12	0.3513 (6)	0.3858 (5)	0.3196 (11)	0.105 (4)	
H12A	0.3135	0.4087	0.3447	0.157*	
H12B	0.3342	0.3578	0.3520	0.157*	
H12C	0.3499	0.3840	0.2365	0.157*	
C13	0.961 (2)	0.3356 (13)	0.107 (3)	0.141 (9)*	0.50
H13A	0.9734	0.3044	0.1013	0.211*	0.50
H13B	0.9908	0.3515	0.0482	0.211*	0.50
H13C	0.9012	0.3400	0.0957	0.211*	0.50
C13'	0.960 (2)	0.3183 (12)	0.184 (3)	0.141 (9)*	0.50
H13D	0.9508	0.2952	0.2394	0.211*	0.50
H13E	0.9999	0.3085	0.1273	0.211*	0.50
H13F	0.9076	0.3253	0.1453	0.211*	0.50
C14	0.989 (3)	0.3235 (16)	0.330 (4)	0.179 (13)*	0.50
H14A	1.0061	0.3383	0.4004	0.269*	0.50
H14B	1.0274	0.2997	0.3142	0.269*	0.50
H14C	0.9333	0.3117	0.3397	0.269*	0.50
C14'	1.035 (3)	0.3434 (16)	0.359 (4)	0.179 (13)*	0.50
H14D	1.0328	0.3692	0.4081	0.269*	0.50
H14E	1.0920	0.3352	0.3462	0.269*	0.50
H14F	1.0058	0.3193	0.3966	0.269*	0.50
C15	1.1257 (14)	0.4301 (11)	0.270 (3)	0.129 (7)*	0.50
H15A	1.1579	0.4549	0.2981	0.194*	0.50
H15B	1.1383	0.4251	0.1896	0.194*	0.50
H15C	1.1398	0.4041	0.3140	0.194*	0.50
C15'	1.1219 (16)	0.3990 (11)	0.344 (3)	0.129 (7)*	0.50
H15D	1.1546	0.4124	0.4054	0.194*	0.50
H15E	1.1509	0.4023	0.2720	0.194*	0.50
H15F	1.1142	0.3680	0.3606	0.194*	0.50
C16	1.0160 (19)	0.4804 (8)	0.340 (2)	0.114 (7)*	0.50
H16A	1.0674	0.4961	0.3568	0.170*	0.50
H16B	0.9878	0.4740	0.4119	0.170*	0.50
H16C	0.9802	0.4984	0.2922	0.170*	0.50
C16'	1.0289 (19)	0.4639 (8)	0.398 (2)	0.114 (7)*	0.50
H16D	1.0755	0.4682	0.4505	0.170*	0.50
H16E	0.9773	0.4636	0.4408	0.170*	0.50
H16F	1.0274	0.4878	0.3427	0.170*	0.50
C17	1.021 (2)	0.3844 (14)	0.026 (3)	0.163 (11)*	0.50
H17A	1.0021	0.3880	−0.0525	0.245*	0.50
H17B	1.0264	0.3533	0.0438	0.245*	0.50
H17C	1.0746	0.3987	0.0364	0.245*	0.50
C17'	1.048 (2)	0.4179 (14)	0.039 (3)	0.163 (11)*	0.50
H17D	1.0404	0.4306	−0.0367	0.245*	0.50
H17E	1.0589	0.3866	0.0316	0.245*	0.50
H17F	1.0958	0.4319	0.0764	0.245*	0.50
C18	0.910 (2)	0.4375 (10)	0.042 (3)	0.132 (8)*	0.50
H18A	0.9126	0.4323	−0.0400	0.197*	0.50
H18B	0.9312	0.4666	0.0592	0.197*	0.50
H18C	0.8525	0.4353	0.0668	0.197*	0.50
C18'	0.918 (2)	0.4601 (10)	0.070 (3)	0.132 (8)*	0.50
H18D	0.9285	0.4669	−0.0100	0.197*	0.50
H18E	0.9273	0.4862	0.1164	0.197*	0.50
H18F	0.8602	0.4507	0.0779	0.197*	0.50
C19	0.946 (3)	0.3996 (11)	0.610 (4)	0.160 (10)*	0.50
H19A	1.0020	0.3967	0.6419	0.240*	0.50
H19B	0.9203	0.4259	0.6402	0.240*	0.50
H19C	0.9491	0.4015	0.5271	0.240*	0.50
C19'	0.833 (2)	0.3884 (13)	0.811 (3)	0.160 (10)*	0.50
H19D	0.8814	0.4020	0.8470	0.240*	0.50
H19E	0.8137	0.3644	0.8584	0.240*	0.50
H19F	0.7894	0.4100	0.8013	0.240*	0.50
C20	0.954 (2)	0.3283 (12)	0.689 (3)	0.165 (11)*	0.50
H20A	1.0089	0.3411	0.7002	0.247*	0.50
H20B	0.9578	0.3037	0.6360	0.247*	0.50
H20C	0.9330	0.3180	0.7620	0.247*	0.50
C20'	0.917 (3)	0.4032 (12)	0.661 (4)	0.165 (11)*	0.50
H20D	0.9474	0.4149	0.7262	0.247*	0.50
H20E	0.8884	0.4269	0.6211	0.247*	0.50
H20F	0.9553	0.3889	0.6089	0.247*	0.50
C21	0.8011 (16)	0.2713 (9)	0.510 (2)	0.092 (5)*	0.50
H21A	0.7845	0.2409	0.5220	0.138*	0.50
H21B	0.8610	0.2726	0.4998	0.138*	0.50
H21C	0.7735	0.2826	0.4415	0.138*	0.50
C21'	0.8366 (17)	0.2732 (9)	0.488 (2)	0.092 (5)*	0.50
H21D	0.8760	0.2490	0.4878	0.138*	0.50
H21E	0.8416	0.2900	0.4181	0.138*	0.50
H21F	0.7804	0.2617	0.4943	0.138*	0.50
C22	0.762 (3)	0.2714 (13)	0.712 (3)	0.171 (11)*	0.50
H22A	0.7528	0.2408	0.6912	0.256*	0.50
H22B	0.7123	0.2826	0.7500	0.256*	0.50
H22C	0.8090	0.2735	0.7643	0.256*	0.50
C22'	0.920 (2)	0.2833 (14)	0.661 (3)	0.171 (11)*	0.50
H22D	0.9425	0.2568	0.6270	0.256*	0.50
H22E	0.8968	0.2763	0.7358	0.256*	0.50
H22F	0.9641	0.3049	0.6709	0.256*	0.50
C23	0.749 (3)	0.3828 (14)	0.834 (2)	0.167 (11)*	0.50
H23A	0.6990	0.3977	0.8603	0.250*	0.50
H23B	0.7968	0.4015	0.8466	0.250*	0.50
H23C	0.7559	0.3556	0.8765	0.250*	0.50
C23'	0.710 (3)	0.2848 (11)	0.740 (3)	0.167 (11)*	0.50
H23D	0.6558	0.2817	0.7737	0.250*	0.50
H23E	0.7528	0.2825	0.7995	0.250*	0.50
H23G	0.7188	0.2617	0.6836	0.250*	0.50
C24	0.6495 (17)	0.3780 (13)	0.699 (4)	0.140 (9)*	0.50
H24A	0.6274	0.3930	0.7653	0.210*	0.50
H24D	0.6240	0.3492	0.6919	0.210*	0.50
H24B	0.6374	0.3950	0.6302	0.210*	0.50
C24'	0.650 (2)	0.3596 (12)	0.708 (4)	0.140 (9)*	0.50
H24C	0.6056	0.3449	0.7491	0.210*	0.50
H24G	0.6277	0.3712	0.6359	0.210*	0.50
H24E	0.6718	0.3836	0.7537	0.210*	0.50

### Atomic displacement parameters (Å^2^)

**Table:**

	*U*^11^	*U*^22^	*U*^33^	*U*^12^	*U*^13^	*U*^23^
W1	0.0533 (2)	0.0542 (2)	0.03251 (17)	−0.00338 (15)	−0.00248 (13)	0.00355 (13)
La1	0.0354 (2)	0.0342 (2)	0.0583 (3)	−0.00025 (17)	−0.00119 (19)	0.00055 (19)
Ag1	0.1111 (7)	0.0849 (6)	0.0349 (4)	0.0012 (5)	−0.0010 (4)	0.0025 (3)
S1	0.0606 (14)	0.112 (2)	0.0514 (13)	−0.0217 (14)	−0.0098 (11)	0.0068 (13)
S2	0.0584 (14)	0.121 (2)	0.0511 (13)	0.0100 (14)	0.0028 (11)	0.0030 (14)
S3	0.0926 (17)	0.0554 (13)	0.0492 (12)	0.0010 (12)	−0.0029 (12)	0.0122 (10)
S4	0.111 (2)	0.0519 (13)	0.0558 (13)	−0.0072 (13)	0.0009 (13)	0.0083 (11)
P1	0.102 (2)	0.0526 (14)	0.0664 (16)	0.0230 (14)	0.0073 (14)	0.0130 (12)
P2	0.0386 (10)	0.0595 (13)	0.0672 (14)	−0.0086 (10)	−0.0023 (10)	−0.0078 (11)
P3	0.0387 (11)	0.0731 (16)	0.0931 (19)	0.0042 (11)	0.0102 (12)	0.0047 (14)
P4	0.098 (2)	0.0694 (17)	0.0616 (15)	0.0005 (15)	−0.0249 (14)	0.0056 (13)
O1	0.130 (4)	0.073 (3)	0.087 (3)	0.012 (3)	−0.001 (3)	−0.018 (2)
O2	0.130 (4)	0.073 (3)	0.087 (3)	0.012 (3)	−0.001 (3)	−0.018 (2)
O3	0.130 (4)	0.073 (3)	0.087 (3)	0.012 (3)	−0.001 (3)	−0.018 (2)
O4	0.082 (3)	0.087 (3)	0.096 (3)	0.007 (2)	−0.011 (2)	−0.037 (2)
O5	0.082 (3)	0.087 (3)	0.096 (3)	0.007 (2)	−0.011 (2)	−0.037 (2)
O6	0.082 (3)	0.087 (3)	0.096 (3)	0.007 (2)	−0.011 (2)	−0.037 (2)
O7	0.078 (4)	0.037 (3)	0.088 (5)	0.002 (3)	−0.006 (3)	0.013 (3)
O8	0.039 (3)	0.060 (4)	0.072 (4)	−0.010 (3)	0.002 (3)	−0.003 (3)
O9	0.036 (3)	0.064 (4)	0.100 (5)	0.002 (3)	0.011 (3)	−0.007 (3)
O10	0.078 (4)	0.056 (4)	0.076 (4)	0.002 (3)	−0.017 (3)	0.016 (3)
N1	0.087 (6)	0.094 (7)	0.090 (7)	0.009 (5)	0.015 (5)	−0.057 (6)
N2	0.110 (7)	0.058 (5)	0.073 (5)	0.025 (5)	−0.031 (5)	−0.028 (4)
N3	0.179 (12)	0.041 (5)	0.164 (11)	−0.014 (6)	0.039 (10)	0.003 (6)
N4	0.171 (11)	0.089 (7)	0.073 (6)	0.031 (7)	0.013 (7)	0.027 (5)
N5	0.178 (12)	0.129 (10)	0.134 (10)	0.096 (10)	0.058 (9)	0.074 (8)
N6	0.068 (5)	0.108 (7)	0.074 (6)	−0.004 (5)	−0.012 (4)	−0.028 (5)
N7	0.063 (5)	0.074 (6)	0.105 (7)	−0.009 (4)	0.009 (5)	0.015 (5)
N8	0.039 (4)	0.087 (6)	0.082 (5)	−0.004 (4)	0.002 (4)	−0.014 (5)
N9	0.076 (7)	0.143 (12)	0.229 (17)	0.000 (7)	0.029 (9)	−0.079 (12)
C7	0.116 (11)	0.203 (18)	0.082 (9)	0.012 (11)	−0.036 (8)	0.002 (10)
C8	0.135 (13)	0.120 (12)	0.179 (16)	−0.018 (10)	−0.036 (12)	−0.078 (12)
C9	0.101 (9)	0.116 (11)	0.100 (9)	−0.023 (8)	0.011 (7)	0.015 (8)
C10	0.107 (10)	0.084 (9)	0.145 (13)	0.007 (8)	0.025 (9)	0.027 (8)
C11	0.074 (7)	0.113 (10)	0.132 (11)	0.005 (7)	0.015 (7)	−0.040 (9)
C12	0.049 (6)	0.149 (12)	0.116 (10)	0.012 (7)	−0.013 (6)	0.005 (9)

### Geometric parameters (Å, °)

**Table:**

W1—S1	2.189 (2)	C3'—H3'C	0.9600
W1—S2	2.196 (3)	C4—H4A	0.9600
W1—S4	2.199 (3)	C4—H4B	0.9600
W1—S3	2.200 (2)	C4—H4C	0.9600
W1—Ag1^i^	2.9561 (8)	C4'—H4'A	0.9600
W1—Ag1	2.9819 (8)	C4'—H4'B	0.9600
La1—O8	2.386 (5)	C4'—H4'C	0.9600
La1—O9	2.390 (5)	C5—H5A	0.9600
La1—O10	2.396 (6)	C5—H5B	0.9600
La1—O7	2.416 (5)	C5—H5C	0.9600
La1—O2	2.598 (8)	C5'—H5'A	0.9600
La1—O1	2.608 (7)	C5'—H5'B	0.9600
La1—O5	2.613 (7)	C5'—H5'C	0.9600
La1—O4	2.614 (7)	C6—H6A	0.9600
La1—N1	2.998 (8)	C6—H6B	0.9600
La1—N2	3.038 (8)	C6—H6C	0.9600
Ag1—S4^ii^	2.496 (2)	C6'—H6'A	0.9600
Ag1—S3^ii^	2.566 (3)	C6'—H6'B	0.9600
Ag1—S2	2.614 (3)	C6'—H6'C	0.9600
Ag1—S1	2.621 (3)	C7—H7A	0.9600
Ag1—W1^ii^	2.9561 (8)	C7—H7B	0.9600
S3—Ag1^i^	2.566 (3)	C7—H7C	0.9600
S4—Ag1^i^	2.496 (2)	C8—H8A	0.9600
P1—O7	1.466 (6)	C8—H8B	0.9600
P1—N4	1.618 (10)	C8—H8C	0.9600
P1—N5	1.627 (12)	C9—H9A	0.9600
P1—N3	1.638 (13)	C9—H9B	0.9600
P2—O8	1.491 (5)	C9—H9C	0.9600
P2—N6	1.608 (8)	C10—H10A	0.9600
P2—N8	1.630 (8)	C10—H10B	0.9600
P2—N7	1.631 (9)	C10—H10C	0.9600
P3—O9	1.491 (6)	C11—H11A	0.9600
P3—N10	1.511 (19)	C11—H11B	0.9600
P3—N10'	1.599 (19)	C11—H11C	0.9600
P3—N9	1.638 (15)	C12—H12A	0.9600
P3—N11	1.65 (2)	C12—H12B	0.9600
P3—N11'	1.68 (2)	C12—H12C	0.9600
P4—O10	1.470 (6)	C13—H13A	0.9600
P4—N13	1.544 (19)	C13—H13B	0.9600
P4—N12'	1.55 (2)	C13—H13C	0.9600
P4—N14'	1.63 (2)	C13'—H13D	0.9600
P4—N14	1.64 (2)	C13'—H13E	0.9600
P4—N12	1.68 (2)	C13'—H13F	0.9600
P4—N13'	1.699 (19)	C14—H14A	0.9600
O1—N1	1.251 (12)	C14—H14B	0.9600
O2—N1	1.252 (12)	C14—H14C	0.9600
O3—N1	1.231 (10)	C14'—H14D	0.9600
O4—N2	1.260 (11)	C14'—H14E	0.9600
O5—N2	1.267 (11)	C14'—H14F	0.9600
O6—N2	1.211 (10)	C15—H15A	0.9600
N3—C1'	1.418 (19)	C15—H15B	0.9600
N3—C2	1.437 (19)	C15—H15C	0.9600
N3—C1	1.456 (19)	C15'—H15D	0.9600
N3—C2'	1.462 (19)	C15'—H15E	0.9600
N4—C3	1.416 (17)	C15'—H15F	0.9600
N4—C3'	1.423 (17)	C16—H16A	0.9600
N4—C4	1.467 (18)	C16—H16B	0.9600
N4—C4'	1.468 (18)	C16—H16C	0.9600
N5—C6'	1.444 (18)	C16'—H16D	0.9600
N5—C6	1.453 (18)	C16'—H16E	0.9600
N5—C5	1.462 (18)	C16'—H16F	0.9600
N5—C5'	1.473 (18)	C17—H17A	0.9600
N6—C7	1.439 (16)	C17—H17B	0.9600
N6—C8	1.473 (16)	C17—H17C	0.9600
N7—C10	1.440 (14)	C17'—H17D	0.9600
N7—C9	1.441 (14)	C17'—H17E	0.9600
N8—C11	1.447 (14)	C17'—H17F	0.9600
N8—C12	1.464 (12)	C18—H18A	0.9600
N9—C13'	1.33 (4)	C18—H18B	0.9600
N9—C14	1.42 (5)	C18—H18C	0.9600
N9—C14'	1.61 (4)	C18'—H18D	0.9600
N9—C13	1.66 (4)	C18'—H18E	0.9600
N10—C16	1.427 (17)	C18'—H18F	0.9600
N10—C15	1.484 (17)	C19—H19A	0.9600
N10'—C15'	1.479 (17)	C19—H19B	0.9600
N10'—C16'	1.481 (17)	C19—H19C	0.9600
N11—C17	1.436 (18)	C19'—H19D	0.9600
N11—C18	1.464 (18)	C19'—H19E	0.9600
N11'—C18'	1.443 (18)	C19'—H19F	0.9600
N11'—C17'	1.458 (18)	C20—H20A	0.9600
N12—C19	1.429 (19)	C20—H20B	0.9600
N12—C20	1.450 (18)	C20—H20C	0.9600
N12'—C19'	1.421 (18)	C20'—H20D	0.9600
N12'—C20'	1.429 (19)	C20'—H20E	0.9600
N12'—N14	1.85 (3)	C20'—H20F	0.9600
N13—C21	1.435 (17)	C21—H21A	0.9600
N13—C22	1.460 (19)	C21—H21B	0.9600
N13'—C21'	1.439 (17)	C21—H21C	0.9600
N13'—C22'	1.459 (18)	C21'—H21D	0.9600
N14—C23	1.440 (18)	C21'—H21E	0.9600
N14—C24	1.457 (18)	C21'—H21F	0.9600
N14'—C24'	1.454 (18)	C22—H22A	0.9600
N14'—C23'	1.456 (18)	C22—H22B	0.9600
C1—H1A	0.9600	C22—H22C	0.9600
C1—H1B	0.9600	C22'—H22D	0.9600
C1—H1C	0.9600	C22'—H22E	0.9600
C1'—H1'A	0.9600	C22'—H22F	0.9600
C1'—H1'B	0.9600	C23—H23A	0.9600
C1'—H1'C	0.9600	C23—H23B	0.9600
C2—H2A	0.9600	C23—H23C	0.9600
C2—H2B	0.9600	C23'—H23D	0.9600
C2—H2C	0.9600	C23'—H23E	0.9600
C2'—H2'A	0.9600	C23'—H23G	0.9600
C2'—H2'B	0.9600	C24—H24A	0.9600
C2'—H2'C	0.9600	C24—H24D	0.9600
C3—H3A	0.9600	C24—H24B	0.9600
C3—H3B	0.9600	C24'—H24C	0.9600
C3—H3C	0.9600	C24'—H24G	0.9600
C3'—H3'A	0.9600	C24'—H24E	0.9600
C3'—H3'B	0.9600		
			
S1—W1—S2	110.48 (10)	H1'A—C1'—H1'C	109.5
S1—W1—S4	108.31 (11)	H1'B—C1'—H1'C	109.5
S2—W1—S4	108.46 (12)	N3—C2—H2A	109.5
S1—W1—S3	108.21 (10)	N3—C2—H2B	109.5
S2—W1—S3	108.31 (10)	H2A—C2—H2B	109.5
S4—W1—S3	113.08 (9)	N3—C2—H2C	109.5
S1—W1—Ag1^i^	124.56 (8)	H2A—C2—H2C	109.5
S2—W1—Ag1^i^	124.95 (7)	H2B—C2—H2C	109.5
S4—W1—Ag1^i^	55.59 (6)	N3—C2'—H2'A	109.5
S3—W1—Ag1^i^	57.48 (7)	N3—C2'—H2'B	109.5
S1—W1—Ag1	58.54 (7)	H2'A—C2'—H2'B	109.5
S2—W1—Ag1	58.30 (7)	N3—C2'—H2'C	109.5
S4—W1—Ag1	100.30 (6)	H2'A—C2'—H2'C	109.5
S3—W1—Ag1	146.63 (7)	H2'B—C2'—H2'C	109.5
Ag1^i^—W1—Ag1	155.891 (14)	N4—C3—H3A	109.5
O8—La1—O9	156.8 (2)	N4—C3—H3B	109.5
O8—La1—O10	92.8 (2)	H3A—C3—H3B	109.5
O9—La1—O10	88.5 (2)	N4—C3—H3C	109.5
O8—La1—O7	94.8 (2)	H3A—C3—H3C	109.5
O9—La1—O7	92.2 (2)	H3B—C3—H3C	109.5
O10—La1—O7	158.6 (2)	N4—C3'—H3'A	109.5
O8—La1—O2	79.6 (2)	N4—C3'—H3'B	109.5
O9—La1—O2	81.0 (3)	H3'A—C3'—H3'B	109.5
O10—La1—O2	126.6 (2)	N4—C3'—H3'C	109.5
O7—La1—O2	74.6 (2)	H3'A—C3'—H3'C	109.5
O8—La1—O1	76.8 (2)	H3'B—C3'—H3'C	109.5
O9—La1—O1	80.9 (2)	N4—C4—H4A	109.5
O10—La1—O1	77.6 (3)	N4—C4—H4B	109.5
O7—La1—O1	123.6 (3)	H4A—C4—H4B	109.5
O2—La1—O1	49.0 (3)	N4—C4—H4C	109.5
O8—La1—O5	76.4 (2)	H4A—C4—H4C	109.5
O9—La1—O5	126.5 (2)	H4B—C4—H4C	109.5
O10—La1—O5	80.1 (2)	N4—C4'—H4'A	109.5
O7—La1—O5	82.4 (2)	N4—C4'—H4'B	109.5
O2—La1—O5	145.0 (3)	H4'A—C4'—H4'B	109.5
O1—La1—O5	143.9 (3)	N4—C4'—H4'C	109.5
O8—La1—O4	125.0 (2)	H4'A—C4'—H4'C	109.5
O9—La1—O4	78.0 (2)	H4'B—C4'—H4'C	109.5
O10—La1—O4	78.5 (2)	N5—C5—H5A	109.5
O7—La1—O4	80.7 (2)	N5—C5—H5B	109.5
O2—La1—O4	146.7 (3)	H5A—C5—H5B	109.5
O1—La1—O4	148.4 (3)	N5—C5—H5C	109.5
O5—La1—O4	48.6 (2)	H5A—C5—H5C	109.5
O8—La1—N1	75.8 (2)	H5B—C5—H5C	109.5
O9—La1—N1	81.2 (2)	N5—C5'—H5'A	109.5
O10—La1—N1	102.2 (3)	N5—C5'—H5'B	109.5
O7—La1—N1	99.1 (3)	H5'A—C5'—H5'B	109.5
O2—La1—N1	24.5 (3)	N5—C5'—H5'C	109.5
O1—La1—N1	24.5 (3)	H5'A—C5'—H5'C	109.5
O5—La1—N1	152.2 (2)	H5'B—C5'—H5'C	109.5
O4—La1—N1	159.2 (3)	N5—C6—H6A	109.5
O8—La1—N2	100.7 (3)	N5—C6—H6B	109.5
O9—La1—N2	102.1 (3)	H6A—C6—H6B	109.5
O10—La1—N2	76.0 (2)	N5—C6—H6C	109.5
O7—La1—N2	82.9 (2)	H6A—C6—H6C	109.5
O2—La1—N2	157.4 (2)	H6B—C6—H6C	109.5
O1—La1—N2	153.4 (3)	N5—C6'—H6'A	109.5
O5—La1—N2	24.5 (2)	N5—C6'—H6'B	109.5
O4—La1—N2	24.3 (2)	H6'A—C6'—H6'B	109.5
N1—La1—N2	176.1 (3)	N5—C6'—H6'C	109.5
S4^ii^—Ag1—S3^ii^	92.94 (8)	H6'A—C6'—H6'C	109.5
S4^ii^—Ag1—S2	118.81 (10)	H6'B—C6'—H6'C	109.5
S3^ii^—Ag1—S2	121.20 (10)	N6—C7—H7A	109.5
S4^ii^—Ag1—S1	119.66 (9)	N6—C7—H7B	109.5
S3^ii^—Ag1—S1	120.22 (10)	H7A—C7—H7B	109.5
S2—Ag1—S1	86.98 (8)	N6—C7—H7C	109.5
S4^ii^—Ag1—W1^ii^	46.65 (6)	H7A—C7—H7C	109.5
S3^ii^—Ag1—W1^ii^	46.29 (5)	H7B—C7—H7C	109.5
S2—Ag1—W1^ii^	136.59 (7)	N6—C8—H8A	109.5
S1—Ag1—W1^ii^	136.41 (6)	N6—C8—H8B	109.5
S4^ii^—Ag1—W1	147.41 (7)	H8A—C8—H8B	109.5
S3^ii^—Ag1—W1	119.66 (6)	N6—C8—H8C	109.5
S2—Ag1—W1	45.64 (6)	H8A—C8—H8C	109.5
S1—Ag1—W1	45.43 (5)	H8B—C8—H8C	109.5
W1^ii^—Ag1—W1	165.95 (3)	N7—C9—H9A	109.5
W1—S1—Ag1	76.03 (8)	N7—C9—H9B	109.5
W1—S2—Ag1	76.06 (8)	H9A—C9—H9B	109.5
W1—S3—Ag1^i^	76.23 (7)	N7—C9—H9C	109.5
W1—S4—Ag1^i^	77.76 (7)	H9A—C9—H9C	109.5
O7—P1—N4	111.3 (5)	H9B—C9—H9C	109.5
O7—P1—N5	110.2 (5)	N7—C10—H10A	109.5
N4—P1—N5	113.4 (6)	N7—C10—H10B	109.5
O7—P1—N3	112.9 (6)	H10A—C10—H10B	109.5
N4—P1—N3	102.4 (7)	N7—C10—H10C	109.5
N5—P1—N3	106.4 (8)	H10A—C10—H10C	109.5
O8—P2—N6	111.6 (4)	H10B—C10—H10C	109.5
O8—P2—N8	108.7 (4)	N8—C11—H11A	109.5
N6—P2—N8	108.9 (4)	N8—C11—H11B	109.5
O8—P2—N7	111.4 (4)	H11A—C11—H11B	109.5
N6—P2—N7	107.1 (5)	N8—C11—H11C	109.5
N8—P2—N7	109.0 (5)	H11A—C11—H11C	109.5
O9—P3—N10	112.5 (8)	H11B—C11—H11C	109.5
O9—P3—N10'	109.7 (7)	N8—C12—H12A	109.5
N10—P3—N10'	31.3 (9)	N8—C12—H12B	109.5
O9—P3—N9	116.7 (5)	H12A—C12—H12B	109.5
N10—P3—N9	119.5 (9)	N8—C12—H12C	109.5
N10'—P3—N9	97.9 (9)	H12A—C12—H12C	109.5
O9—P3—N11	109.9 (8)	H12B—C12—H12C	109.5
N10—P3—N11	110.2 (11)	N9—C13—H13A	109.5
N10'—P3—N11	134.4 (11)	N9—C13—H13B	109.5
N9—P3—N11	83.9 (10)	H13A—C13—H13B	109.5
O9—P3—N11'	109.7 (8)	N9—C13—H13C	109.5
N10—P3—N11'	90.4 (11)	H13A—C13—H13C	109.5
N10'—P3—N11'	119.1 (10)	H13B—C13—H13C	109.5
N9—P3—N11'	103.6 (10)	N9—C13'—H13D	109.5
N11—P3—N11'	22.8 (10)	N9—C13'—H13E	109.5
O10—P4—N13	110.3 (8)	H13D—C13'—H13E	109.5
O10—P4—N12'	121.0 (9)	N9—C13'—H13F	109.5
N13—P4—N12'	125.9 (11)	H13D—C13'—H13F	109.5
O10—P4—N14'	113.7 (9)	H13E—C13'—H13F	109.5
N13—P4—N14'	60.4 (10)	N9—C14—H14A	109.5
N12'—P4—N14'	108.7 (12)	N9—C14—H14B	109.5
O10—P4—N14	105.9 (9)	H14A—C14—H14B	109.5
N13—P4—N14	111.7 (11)	N9—C14—H14C	109.5
N12'—P4—N14	70.8 (12)	H14A—C14—H14C	109.5
N14'—P4—N14	52.4 (10)	H14B—C14—H14C	109.5
O10—P4—N12	107.3 (8)	N9—C14'—H14D	109.5
N13—P4—N12	115.3 (11)	N9—C14'—H14E	109.5
N12'—P4—N12	35.3 (10)	H14D—C14'—H14E	109.5
N14'—P4—N12	137.4 (11)	N9—C14'—H14F	109.5
N14—P4—N12	105.9 (11)	H14D—C14'—H14F	109.5
O10—P4—N13'	109.3 (7)	H14E—C14'—H14F	109.5
N13—P4—N13'	44.9 (8)	N10—C15—H15A	109.5
N12'—P4—N13'	99.4 (11)	N10—C15—H15B	109.5
N14'—P4—N13'	102.2 (10)	H15A—C15—H15B	109.5
N14—P4—N13'	143.2 (10)	N10—C15—H15C	109.5
N12—P4—N13'	73.8 (10)	H15A—C15—H15C	109.5
N1—O1—La1	95.5 (6)	H15B—C15—H15C	109.5
N1—O2—La1	95.9 (6)	N10'—C15'—H15D	109.5
N2—O4—La1	97.0 (5)	N10'—C15'—H15E	109.5
N2—O5—La1	96.9 (6)	H15D—C15'—H15E	109.5
P1—O7—La1	171.3 (4)	N10'—C15'—H15F	109.5
P2—O8—La1	167.2 (4)	H15D—C15'—H15F	109.5
P3—O9—La1	162.0 (4)	H15E—C15'—H15F	109.5
P4—O10—La1	169.1 (4)	N10—C16—H16A	109.5
O3—N1—O1	121.2 (11)	N10—C16—H16B	109.5
O3—N1—O2	119.5 (11)	H16A—C16—H16B	109.5
O1—N1—O2	119.3 (8)	N10—C16—H16C	109.5
O3—N1—La1	173.8 (7)	H16A—C16—H16C	109.5
O1—N1—La1	60.0 (5)	H16B—C16—H16C	109.5
O2—N1—La1	59.5 (5)	N10'—C16'—H16D	109.5
O6—N2—O4	123.2 (9)	N10'—C16'—H16E	109.5
O6—N2—O5	120.2 (10)	H16D—C16'—H16E	109.5
O4—N2—O5	116.6 (8)	N10'—C16'—H16F	109.5
O6—N2—La1	171.5 (7)	H16D—C16'—H16F	109.5
O4—N2—La1	58.7 (4)	H16E—C16'—H16F	109.5
O5—N2—La1	58.7 (5)	N11—C17—H17A	109.5
C1'—N3—C2	25 (3)	N11—C17—H17B	109.5
C1'—N3—C1	90 (3)	H17A—C17—H17B	109.5
C2—N3—C1	109 (3)	N11—C17—H17C	109.5
C1'—N3—C2'	95 (3)	H17A—C17—H17C	109.5
C2—N3—C2'	75 (3)	H17B—C17—H17C	109.5
C1—N3—C2'	104 (3)	N11'—C17'—H17D	109.5
C1'—N3—P1	130 (3)	N11'—C17'—H17E	109.5
C2—N3—P1	122 (3)	H17D—C17'—H17E	109.5
C1—N3—P1	125 (2)	N11'—C17'—H17F	109.5
C2'—N3—P1	106 (2)	H17D—C17'—H17F	109.5
C3—N4—C3'	29.8 (17)	H17E—C17'—H17F	109.5
C3—N4—C4	116 (2)	N11—C18—H18A	109.5
C3'—N4—C4	126 (2)	N11—C18—H18B	109.5
C3—N4—C4'	101 (2)	H18A—C18—H18B	109.5
C3'—N4—C4'	101 (2)	N11—C18—H18C	109.5
C4—N4—C4'	27 (2)	H18A—C18—H18C	109.5
C3—N4—P1	128.9 (15)	H18B—C18—H18C	109.5
C3'—N4—P1	118.6 (15)	N11'—C18'—H18D	109.5
C4—N4—P1	112.8 (19)	N11'—C18'—H18E	109.5
C4'—N4—P1	129.5 (19)	H18D—C18'—H18E	109.5
C6'—N5—C6	16 (3)	N11'—C18'—H18F	109.5
C6'—N5—C5	101 (3)	H18D—C18'—H18F	109.5
C6—N5—C5	102 (3)	H18E—C18'—H18F	109.5
C6'—N5—C5'	129 (3)	N12—C19—H19A	109.5
C6—N5—C5'	121 (3)	N12—C19—H19B	109.5
C5—N5—C5'	37.1 (17)	H19A—C19—H19B	109.5
C6'—N5—P1	119 (2)	N12—C19—H19C	109.5
C6—N5—P1	126 (2)	H19A—C19—H19C	109.5
C5—N5—P1	129.0 (17)	H19B—C19—H19C	109.5
C5'—N5—P1	111.9 (18)	N12'—C19'—H19D	109.5
C7—N6—C8	117.2 (11)	N12'—C19'—H19E	109.5
C7—N6—P2	119.2 (9)	H19D—C19'—H19E	109.5
C8—N6—P2	123.2 (10)	N12'—C19'—H19F	109.5
C10—N7—C9	113.8 (10)	H19D—C19'—H19F	109.5
C10—N7—P2	120.4 (8)	H19E—C19'—H19F	109.5
C9—N7—P2	125.7 (8)	N12—C20—H20A	109.5
C11—N8—C12	115.6 (9)	N12—C20—H20B	109.5
C11—N8—P2	121.1 (7)	H20A—C20—H20B	109.5
C12—N8—P2	121.4 (8)	N12—C20—H20C	109.5
C13'—N9—C14	80 (3)	H20A—C20—H20C	109.5
C13'—N9—C14'	113 (3)	H20B—C20—H20C	109.5
C14—N9—C14'	38 (2)	N12'—C20'—H20D	109.5
C13'—N9—P3	138 (2)	N12'—C20'—H20E	109.5
C14—N9—P3	126 (2)	H20D—C20'—H20E	109.5
C14'—N9—P3	103.9 (19)	N12'—C20'—H20F	109.5
C13'—N9—C13	38.1 (18)	H20D—C20'—H20F	109.5
C14—N9—C13	118 (3)	H20E—C20'—H20F	109.5
C14'—N9—C13	147 (2)	N13—C21—H21A	109.5
P3—N9—C13	109.1 (18)	N13—C21—H21B	109.5
C16—N10—C15	115 (2)	H21A—C21—H21B	109.5
C16—N10—P3	122.2 (18)	N13—C21—H21C	109.5
C15—N10—P3	122.8 (18)	H21A—C21—H21C	109.5
C15'—N10'—C16'	117 (2)	H21B—C21—H21C	109.5
C15'—N10'—P3	124.0 (18)	N13'—C21'—H21D	109.5
C16'—N10'—P3	117.4 (17)	N13'—C21'—H21E	109.5
C17—N11—C18	111 (3)	H21D—C21'—H21E	109.5
C17—N11—P3	127 (2)	N13'—C21'—H21F	109.5
C18—N11—P3	118 (2)	H21D—C21'—H21F	109.5
C18'—N11'—C17'	116 (3)	H21E—C21'—H21F	109.5
C18'—N11'—P3	119 (2)	N13—C22—H22A	109.5
C17'—N11'—P3	122 (2)	N13—C22—H22B	109.5
C19—N12—C20	108 (3)	H22A—C22—H22B	109.5
C19—N12—P4	131 (2)	N13—C22—H22C	109.5
C20—N12—P4	120 (2)	H22A—C22—H22C	109.5
C19'—N12'—C20'	103 (3)	H22B—C22—H22C	109.5
C19'—N12'—P4	126 (2)	N13'—C22'—H22D	109.5
C20'—N12'—P4	121 (3)	N13'—C22'—H22E	109.5
C19'—N12'—N14	70 (2)	H22D—C22'—H22E	109.5
C20'—N12'—N14	133 (3)	N13'—C22'—H22F	109.5
P4—N12'—N14	56.8 (10)	H22D—C22'—H22F	109.5
C21—N13—C22	114 (2)	H22E—C22'—H22F	109.5
C21—N13—P4	121.2 (18)	N14—C23—H23A	109.5
C22—N13—P4	122 (2)	N14—C23—H23B	109.5
C21'—N13'—C22'	112 (2)	H23A—C23—H23B	109.5
C21'—N13'—P4	121.8 (17)	N14—C23—H23C	109.5
C22'—N13'—P4	126 (2)	H23A—C23—H23C	109.5
C23—N14—C24	100 (3)	H23B—C23—H23C	109.5
C23—N14—P4	137 (2)	N14'—C23'—H23D	109.5
C24—N14—P4	121 (2)	N14'—C23'—H23E	109.5
C23—N14—N12'	90 (2)	H23D—C23'—H23E	109.5
C24—N14—N12'	169 (3)	N14'—C23'—H23G	109.5
P4—N14—N12'	52.3 (10)	H23D—C23'—H23G	109.5
C24'—N14'—C23'	116 (3)	H23E—C23'—H23G	109.5
C24'—N14'—P4	115 (2)	N14—C24—H24A	109.5
C23'—N14'—P4	127 (2)	N14—C24—H24D	109.5
N3—C1—H1A	109.5	H24A—C24—H24D	109.5
N3—C1—H1B	109.5	N14—C24—H24B	109.5
H1A—C1—H1B	109.5	H24A—C24—H24B	109.5
N3—C1—H1C	109.5	H24D—C24—H24B	109.5
H1A—C1—H1C	109.5	N14'—C24'—H24C	109.5
H1B—C1—H1C	109.5	N14'—C24'—H24G	109.5
N3—C1'—H1'A	109.5	H24C—C24'—H24G	109.5
N3—C1'—H1'B	109.5	N14'—C24'—H24E	109.5
H1'A—C1'—H1'B	109.5	H24C—C24'—H24E	109.5
N3—C1'—H1'C	109.5	H24G—C24'—H24E	109.5

Symmetry codes: (i) *x*, −*y*+1/2, *z*+1/2; (ii) *x*, −*y*+1/2, *z*−1/2.
